# Food Insecurity in Delaware: A Triangulation of Spatial Data Sources

**DOI:** 10.5888/pcd18.200555

**Published:** 2021-08-19

**Authors:** Cecelia Harrison, Madeline Brooks, Jennifer N. Goldstein, Mia Papas

**Affiliations:** 1 ChristianaCare, Value Institute, Newark, Delaware

**Figure:**
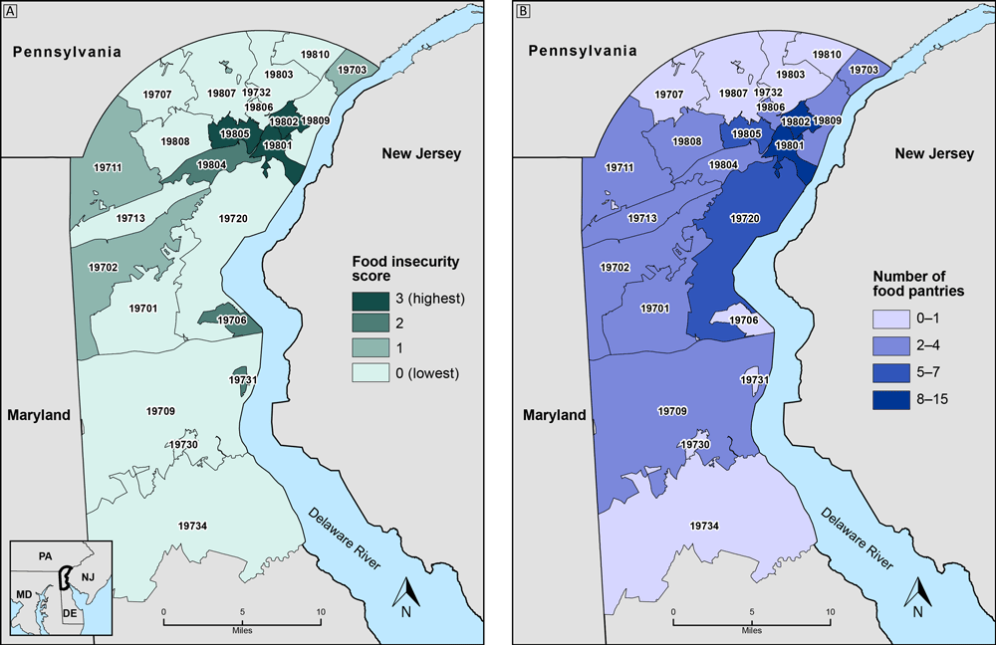
Distribution of food insecurity summary scores (Map A) and food pantries (Map B) in New Castle County, Delaware, by zip code. Inset shows the location of New Castle County. The burden of food insecurity is highest in three zip codes (19801, 19802, and 19805) in northeastern New Castle County that also have relatively high numbers of food pantries. Food insecurity burden was estimated by using a summary score that combined patient screening data and American Community Survey data for household poverty and Supplemental Nutrition Assistance Program (SNAP) participation. Data sources: ChristianaCare, 2018–2019 (1); US Census Bureau American Community Survey 5-Year Estimates, 2013–2017 (2); 2-1-1 Delaware, 2019 (3).

## Background

Medical care accounts for a small fraction of the variability in preventable mortality in the US (4). Health promotion and disease prevention can be achieved primarily through a focus on social determinants. Food insecurity, defined as limited or uncertain access to food, is a social determinant of health that should be accounted for in population health strategies. Eleven percent of the US population and 12.6% of Delawareans are food insecure (5), with a higher prevalence evident among racial minorities, low-income households, and people with chronic disease (6–8). Identification of food insecurity can trigger the delivery of interventions that can prevent chronic disease and improve health. To be effective, interventions must consider where patients reside, because previous work has shown that screening for food insecurity does not necessarily facilitate access to food resources (9). Few studies have examined the spatial distribution of food insecurity at local levels (10). We sought to identify zip codes with high burdens of food insecurity and relatively few food resources.

We conducted a food insecurity screening survey in ChristianaCare primary care clinics in New Castle County, Delaware, from 2018–2019. Because the screening data from the survey may not be representative of the spatial distribution of the general population, incorporating other data sources can “triangulate” or corroborate spatial patterns of health outcomes or need. This approach is especially useful where small-area data for outcomes such as food insecurity are not available. We demonstrate an approach in which providers can employ multiple spatial data sources to identify where needs are prevalent and connect patients in those areas to services.

## Data and Methods

We conducted a cross-sectional survey of adult patients from 4 ChristianaCare primary care clinics in New Castle County, Delaware, from 2018 through 2019 (1). Research assistants read survey questions to participants in examination rooms and collected data. The prevalence of household food insecurity was determined according to the 18-item USDA Household Food Insecurity Scale (11). Food insecurity was treated as a binary variable with a raw score of ≤3 indicating food insecurity; raw scores range from 0 (high food security) to 18 (very low food security) (11). Demographic and clinical data were also collected. Screened patients were aggregated to their home zip codes to create ratios of food insecure to food secure patients, adjusting for geographic variation based on where patients resided. Zip code data were obtained from the US Census Bureau’s American Community Survey for the percentage of households below federal poverty level and the percentage of households receiving Supplemental Nutrition Assistance Program (SNAP) benefits (2,12), These measures were chosen as population-level indicators of food insecurity because poverty has been associated with food insecurity and many SNAP recipients remain food insecure despite this assistance (8,12,13). A summary score (ranging from 0–3) was created to identify zip codes in the top quartiles for food insecurity ratios, household poverty levels, and household SNAP participation. A score of 3 indicates a zip code with the highest rank in all categories, representing high expected levels of food insecurity. A directory of food pantries was created and mapped to examine the zip code distribution of community-based nutrition resources. This directory included state service centers, nonprofit organizations, and houses of worship that provide emergency food support (3). Because these institutions vary in the number of people they serve, they were used not to indicate need but to describe their spatial distribution in relation to the zip code score, indicating a need for food resources. We used ArcGIS 10.6 (Esri) for data integration and mapping.

## Highlights

Approximately 18% of patients (52/295) were food insecure. Of 29 county zip codes, 10 zip code summary scores (34%) ranked in the top quartile for either food insecurity, household poverty, or household SNAP participation. Three zip codes — 19801, 19802, and 19805 — located in the city of Wilmington were in the top quartiles for all 3 indicators. More than a third of food pantries (38%) were located in only 2 zip codes (19801 and 19802), which contained about 8% of the county population. Fewer food resources were present in many zip code areas with higher levels of food insecurity, such as 19706, as measured by having 1 or 2 indicators of food insecurity burden.

## Action

Approximately 18% of the population screened in our sample was affected by food insecurity, and 30% of those affected lived in zip codes 19801 and 19802. The co-location of food pantries in areas with high levels of food insecurity raises questions about how health care systems can facilitate access to nutrition resources for their food-insecure patients. Although it has been shown that food pantry use can be infrequent and serve as a temporary solution for supplementary nutrition, emergency food aid is often one of the only reliable sources of nutrition for food-insecure people (14–16). Therefore, it is imperative that health care systems employ strategies to facilitate access to nutritional resources (14). Common strategies include integrating universal screenings into clinical workflows, involving social workers or case managers in patient care for connection to community and federal resources, referring patients to food resources, and giving referrals to food pantries (14).

As health care systems collect patient-level data on social needs, they must consider the context of social and built environments by using relevant population-level data on socioeconomic status and geographic access to services. Using multiple data sources to conduct small area analyses, such as at the zip code level, allows health care systems to better identify where specific needs are prevalent and refer patients to nearby resources to ensure that distance is not a barrier. Systems are then better equipped to offer patients local interventions while identifying areas of high need that warrant further investment in social resources, such as nutrition support. In these ways, health care systems can leverage spatial data to address patient needs while increasing their capacity to serve the needs of the greater population.

Food insecurity is a social determinant of health that needs to be understood within the greater social and environmental context. Using multiple spatial data sources supports health care systems in partnering with community-based organizations and designing interventions tailored to their populations. These strategies will help to ameliorate the effects of food insecurity, prevent chronic disease, and enhance the health of populations.
